# Improvements

**Published:** 1878-11

**Authors:** 


### IMPROVEMENTS.

If improvements indicate prosperity, then at least two of our dental colleges are on the way to success, viz: the Ohio College of Dental Surgery and the Dental College of the University of Michigan. The former has undergone a thorough renovation from top to bottom. The clinic room has been enlarged to about three times its former capacity, and is well lighted and arranged for the purpose designed. The building was originally built for a dental college, and it is now, as improved, well adapted in all particulars for the purpose.

The dental college at Ann Arbor is being enlarged to more than double its present capacity, by the addition of a substantial brick building sixty-two feet by twenty-four, two stories high. The first story is designed for a laboratory, and the second for a clinic room; these will be supplied with every needed facility for the work to be done. This will give ample accommodation for a class of one hundred.

Such facts as these show a growing interest on the subject of dental education, and argues well for the future.

